# Genomic characterization of vancomycin-resistant *Enterococcus faecium* clonal complex 17 isolated from urine in tertiary hospitals in Northeastern Thailand

**DOI:** 10.3389/fmicb.2023.1278835

**Published:** 2024-01-19

**Authors:** Peechanika Chopjitt, Parichart Boueroy, Piroon Jenjaroenpun, Thidathip Wongsurawat, Rujirat Hatrongjit, Anusak Kerdsin, Nuchsupha Sunthamala

**Affiliations:** ^1^Faculty of Public Health, Kasetsart University Chalermphrakiat Sakon Nakhon Province Campus, Sakon Nakhon, Thailand; ^2^Division of Medical Bioinformatics, Department of Research, Faculty of Medicine Siriraj Hospital, Mahidol University, Bangkok, Thailand; ^3^Faculty of Science and Engineering, Kasetsart University Chalermphrakiat Sakon Nakhon Province Campus, Sakon Nakhon, Thailand; ^4^Department of Biology, Faculty of Science, Mahasarakham University, Kantharawichai, Thailand

**Keywords:** antimicrobial resistance gene, *Enterococcus faecium*, urine, whole genome sequencing, vancomycin-resistant

## Abstract

Vancomycin-resistant Enterococci (VREs) have increasingly become a major nosocomial pathogen worldwide, earning high-priority category from the World Health Organization (WHO) due to their antibiotic resistance. Among VREs, vancomycin-resistant *Enterococcus faecium* (VREfm) is particularly concerning, frequently isolated and resistant to many antibiotics used in hospital-acquired infections. This study investigated VREfm isolates from rural tertiary hospitals in Northeastern Thailand based both antibiotic susceptibility testing and whole-genome sequencing. All isolates showed resistance to vancomycin, ampicillin, erythromycin, tetracycline, ciprofloxacin, norfloxacin, and rifampin. Nitrofurantoin and tigecycline resistance were also observed in nearly all isolates. Conversely, all isolates remained susceptible to chloramphenicol, daptomycin, and linezolid. Genomic characterization revealed that all VREfm isolates belonged to clonal complex 17 (CC17), primarily consisting of sequence type (ST) 80, followed by ST17, ST761, and ST117. Additionally, all isolates harbored numerous antimicrobial-resistant genes, including *vanA, tet(L), tet(M), aac(6′)-li, ant(6)-Ia, aph(3′)-III, aac(6′)-aph(2″), aph(2″)-la, ant(9)-la, erm(B), msr(C), erm(T), erm(A), fosB, dfrG, and* cfr*(B)*. Notably, all isolates contained virulence genes, for collagen adhesin (*acm*) and cell wall adhesin (*efafm*), while *hylEfm* (glycosyl hydrolase) was detected in VREfm ST80. This study provided important information for understanding the genomic features of VREfm isolated from urine.

## 1 Introduction

*Enterococci* are a Gram-positive bacterium, facultatively anaerobic, and widely distributed in nature and in the digestive tracts of humans and animals. They are the second leading cause of healthcare-associated infection and an important pathogen of urethral infection, soft tissue infection, sepsis, and meningitis (Sun et al., 2019). Among these Enterococci, *Enterococcus faecalis* and *Enterococcus faecium* are clinically the most important, especially *E. faecium*, which is nosocomial pathogen responsible for about 95% of human enterococcal infection and a leading cause of hospital-acquired and multidrug-resistant infection (Ahmed and Baptiste, 2018).

Urinary tract infection (UTI) is the most common nosocomial infection caused by this organism (Fallah et al., 2017), and it is of concern due to the limited availability of antimicrobial therapeutic options (Gozalan et al., 2015; Benamrouche et al., 2021). The emergence of multidrug-resistant (MDR) *E. faecium*, particularly VREfm, is an important health concern that has led to high morbidity and mortality in hospitalized patients (Li et al., 2022). Furthermore, VREfm CC17 can spread within hospitals as well as between regions or countries (Akpaka et al., 2017; Gao et al., 2018).

In addition, the WHO has included VREfm in a high-priority list of 12 resistant-bacteria that pose the greatest threat to human health (Tacconelli et al., 2018). In Asia, the prevalence of VREfm accounts for 22.4% of reported cases and is higher than in European countries but lower than in the USA (Shrestha et al., 2021). According to Thailand’s National Antimicrobial Resistance Surveillance Center, the prevalence of *E. faecium* increased from 0.7 to 6.9% between 2012 and 2020 (National Antimicrobial Resistance Surveillance Thailand, 2022). Treatment options for invasive VREfm infections are very limited, resulting in high mortality (Linden, 2002). Vancomycin resistance determinants due to the *vanA* and *vanB* genes are frequently reported globally in VRE, including in *E. faecium* clinical isolates. In Thailand, there have been few studies of the molecular epidemiological characteristics of clinical isolates with respect to the prevalence of genotypes, virulence factors, and the antimicrobial resistance profile of VREfm. Therefore, we aimed to characterize the phenotypic and genotypic resistance profile of 16 VREfm strains isolated from urine samples obtained from tertiary hospitals in northeast Thailand.

## 2 Materials and methods

### 2.1 Bacterial identification

In total, 16 non-duplicate urinary VREfm stains were collected through 2016–2020 from three 500–800-bed tertiary hospitals in Thailand. A total of 1,507 urine samples were collected including hospital A (*n* = 462), hospital B (*n* = 378), and hospital C (*n* = 667). Each isolate of VREfm were cultured on sheep blood agar (HiMedia Laboratories Pvt. Ltd., Nashik, India), followed by incubation for 24 h at 37°C. The colonies with typical enterococcal morphological characteristics were first identified based on Gram staining and standard biochemical tests, including arabinose utilization, growth in 6.5% NaCl, bile esculin degradation, and pyrrolidonyl *β*-naphthylamide (PYR) degradation (Saenhom et al., 2022). All isolates were confirmed by species-specific multiplex polymerase chain reaction (PCR), enterococcal superoxide dismutase (sodA) gene is the identification marker according to previously described by Jackson et al. (2004). Each isolate was stored in a freezer at −80°C.

### 2.2 Multiplex polymerase chain reaction

DNA was extracted from the presumptive VREfm isolates using a heat-lysis method (Liu et al., 2002). A few colonies of each bacterium were resuspended in 20 μL of lysis buffer and heated at 95°C for 20 min. Then, 180 μL sterile deionized (DI) water was added into the lysis buffer and DNA solution and stored at −20°C. The species-specific and the presence of the vancomycin resistance genes *vanA, vanB*, and *vanC* were determined based on our multiplex PCR method, using primers described elsewhere (Pérez-Hernández et al., 2002; Jackson et al., 2004). The total reaction was carried out in a 25 μL of mixture, composed of 12.5 μL of 2X JumpStart^™^ REDTaq^®^ ReadyMix^™^ Reaction Mix (Sigma-Aldrich Co. LLC, MO, United States), 0.5 μM of each forward and reverse primer solution, 2 μl of DNA sample, and DI water to complete the final volume. The procedure consisted of an initial denaturation at 95°C for 4 min, followed by 30 cycles of denaturation (95°C, 30 s), annealing (55°C, 1 min) and extension (72°C, 1 min), with a final extension step (72°C, 7 min). The amplified DNA was separated using submarine gel electrophoresis, stained with ethidium bromide, and visualized under UV transillumination (SynGene; Cambridge, UK). DNA molecular weight marker [DNA Ladder (Thermo Scientific; Vilnius, Lithuania)] was used as the standard. *Enterococcus faecium* ATCC 19434 was used as the positive control strain.

### 2.3 Antimicrobial susceptibility testing

The susceptibility testing for these VREfm isolates was determined for ampicillin (AM, 10 μg), vancomycin (VA, 30 μg), erythromycin (E, 15 μg), tetracycline (TE, 30 μg), ciprofloxacin (CIP, 5 μg), norfloxacin (NX, 10 μg), fosfomycin (FOS, 200 μg), nitrofurantoin (FM, 300 μg), chloramphenicol (C, 30 μg), and rifampin (RA, 5 μg) using the disk diffusion method on Mueller-Hinton agar (bioMerieux; France). Daptomycin, teicoplanin, and linezolid were applied using an E-test (Liofilchem S.r.l.; Italy). The results were interpreted according to the Clinical and Laboratory Standards Institute (CLSI) guidelines (CLSI, 2022). The broth microdilution method was used to test the minimum inhibitory concentration (MIC) of tigecycline, according to European Committee on Antimicrobial Susceptibility Testing (EUCAST, 2021). *E. faecium* ATCC 29212 and *Staphylococcus aureus* ATCC25923 were used as quality controls.

### 2.4 Whole-genomes sequencing and bioinformatic analysis

Bacterial DNA was extracted using a ZymoBIOMIC DNA miniprep Kit (Zymo Research; CA, United States) according to the manufacture’s procedure. The DNA concentration and purity were investigated using a Nanopore 2000 spectrophotometer (Thermo Scientific, DE, United States). Restriction enzymes were used to digest genomic DNA. The digested DNA was ligated to an adaptor and the size distribution of the final PCR-amplified library fragments was investigated. DNA libraries were prepared using the MGIEasy FS DNA Library Prep Set (MGI Tech Co., Ltd., SZ, China). The libraries were quantified using a Qubit 2.0 Fluorometer (Invitrogen, CA, United States) and sequenced using an MGISEQ-2000RS platform (MGI Tech Co., Ltd., SZ, China) with a 150 bp paired-end. A raw data quality check was conducted using FastQC (version 0.11.9).^1^ The Unicycler v0.5.0 software was used to *de novo* assemble a total of raw readings from each sample (Lang et al., 2021). The ST was identified using the MLST 2.0 program (Larsen et al., 2012). Antibiotic resistance genes were identified based on the Comprehensive Antibiotic Resistance Database (Alcock et al., 2023) and the ResFinder4.1 software (Bortolaia et al., 2020). Plasmid replicon and virulence genes were analyzed using the PlasmidFinder2.1 (Carattoli and Hasman, 2020), and VirulenceFinder2.0 (Malberg Tetzschner et al., 2020) programs. Comprehensive genomic analysis was undertaken using the BacWGSTdb server,^2^ which identified the closest isolates bases on small (1–100) single nucleotide polymorphism (SNP) with 16 *E. faecium* strains in our study, currently deposited in the GenBank database (Feng et al., 2021). We found 23 whole-genome sequences closely related to our *E. faecium* strains were downloaded from the GenBank database to produce reconstructed phylogenetic trees based on the web server Reference Sequence Alignment-based Phylogeny (REALPHY) builder^3^ (Bertels et al., 2014). A goeBURST analysis for sequence types was performed using the PHYLOViZ 2.0 program (Francisco et al., 2012). The phylogenetic tree was visualized using the Interactive Tree of Life (iTOL)^4^ (Letunic and Bork, 2021).

Pangenome analysis were performed using Roary software (Page et al., 2015). The software clustered the genomes based on the genes each strain carried. Base on the distribution of each gene among the strains, the genes were divided into core genes and accessory genes. Core genes were defined as those carried by 99% or more of the strains. With the GFF files from Prokka annotation, the software produced a multi-FASTA alignment of core genes using MAFFT with --mafft” option and a presence/absence matrix for the genes. The genes were grouped into “core,” “shell,” and cloud categories for the analysis, corresponding to their presence in 99%, 10–99%, and less than 10% of genomes analyzed, respectively. A graphic representation of pangenome results was prepared using the roary_plots.py script provide on the Roary website.

### 2.5 Data availability statement

The assembled genomic sequences were deposited in the NCBI Genbank Database under the Bioproject accession number PRJNA1002621.

## 3 Results and discussion

The 13 VREfm stains were collected from hospital A, two stains form hospital B, and one isolate from hospital C. Antimicrobial susceptibility among the VREfm isolates is shown in Table 1. All isolates carried the *van*A gene and they were classified as MDR bacteria, with resistance to 100% of vancomycin, ampicillin, erythromycin, tetracycline, ciprofloxacin, and rifampin. Almost all VREfm were resistant to nitrofurantoin (93.75%) and tigecycline (81.25%). Additionally, all VREfm isolates were completely susceptible to chloramphenicol, daptomycin, and linezolid, suggesting that these three antibiotics could be alternative choices for treatment. Other studies reported that all VREs were resistant to ampicillin but susceptible to linezolid (Gozalan et al., 2015; Yang et al., 2015; Rao et al., 2021). Linezolid is the only antibiotic having US Food and Drug Administration approval for the treatment of VRE bacteremia (Miller et al., 2020). Linezolid or oxazolidinones resistance presents a significant concern. In a study conducted by Miller and colleague, they identified two transmissible genes, *cfr* and *optrA,* associated with oxazolidinone resistance in VREs (Miller et al., 2020). In our current study, *optrA* genes were not detected, however, *cfr*(B) was found in four VREfm isolates (25%), specifically strains C1380, C1382, and C2633 (belonging to ST17) as well as AMR0099 ST761.

**Table 1:** Antimicrobial susceptibility profiles of 16 VERfm strains.

ID	Accession No.	ST	Source	Disk diffusion assay										MIC			
				AM	E	TE	CIP	NX	FM	C	RA	FOS	VA	DAP	TEC	LZD	TGC
C1380	JAVRBD000000000	17	Hospital A	R	R	R	R	R	R	S	R	I	R	SDD	S	S	R
C1382	JAVRBC000000000	17	Hospital A	R	R	R	R	R	R	S	R	S	R	SDD	S	S	S
AMR0098	JAVRAS000000000	17	Hospital A	R	R	R	R	R	R	S	R	R	R	SDD	I	S	R
C2633	JAVRAO000000000	17	Hospital B	R	R	R	R	R	R	S	R	I	R	SDD	S	S	R
C2634	JAVRAN000000000	17	Hospital B	R	R	R	R	R	R	S	R	S	R	SDD	I	S	R
C1117	JAVRBE000000000	80	Hospital A	R	R	R	R	R	R	S	R	S	R	SDD	S	S	R
C1852	JAVRBB000000000	80	Hospital A	R	R	R	R	R	R	S	R	S	R	SDD	S	S	R
C1858	JAVRAZ000000000	80	Hospital A	R	R	R	R	R	R	S	R	S	R	SDD	I	S	R
C1877	JAVRAW000000000	80	Hospital A	R	R	R	R	R	R	S	R	S	R	SDD	I	S	R
C2225	JAVRAU000000000	80	Hospital A	R	R	R	R	R	R	S	R	S	R	SDD	I	S	S
AMR0096	JAVRAT000000000	80	Hospital A	R	R	R	R	R	R	S	R	R	R	SDD	I	S	S
C2355	JAVRAP000000000	80	Hospital C	R	R	R	R	R	R	S	R	I	R	SDD	R	S	R
AMR114	JAVRAQ000000000	117	Hospital A	R	R	R	R	R	R	S	R	I	R	SDD	I	S	R
C1876	JAVRAX000000000	761	Hospital A	R	R	R	R	R	R	S	R	R	R	SDD	I	S	R
C1981	JAVRAV000000000	761	Hospital A	R	R	R	R	R	R	S	R	I	R	SDD	I	S	R
AMR0099	JAVRAR000000000	761	Hospital A	R	R	R	R	R	R	S	R	I	R	SDD	I	S	R

*ST, sequence type.

**AM, Ampicillin; E, Erythromycin; TE, Tetracyclin; CIP, Ciprofloxacin; NX, Norfloxacin; FM, Nitrofurantoin; C, Chloramphenicol; RA, Rifampin; FOS, Fosfomycin; VA, Vancomycin; DAP, Daptomycin; TEC, Teicoplanin; LZD, Linezolid; TGC, Tigecycline.

***R, resistance; I, intermediate; S, susceptible; SDD, susceptible-dose dependent.

****MIC breakpoints (μg/mL) according to CLSI (2022) for Daptomycin, ≤ 4 = SDD, ≥8 = R, Teicoplanin, ≤ 8 = S, =16 = I, ≥32 = R, Linezolid, ≤ 2 = S, =4 = I, ≥8 = R.

*****MIC breakpoints for tigecycline according to EUCAST (2021) were 0.25 mg/L.

Cfr, known for conferring chloramphenicol-florfenicol resistance, encode the *S*-adenosylmethionine (SAM) enzyme responsible for methylates the adenine nucleotide at position 2503 of the 23S rRNA. This modification results in resistance not only to oxazolidanones but also to phenicols, pleuromutilins, lincosamides, and streptogramin A (Miller et al., 2020). It’s noteworthy that all 16 VREfms in our study displayed complete susceptible to chloramphenicol and linezolid. Although previous studies, such as those by Liu and colleague and Deshpande and colleague, have reported the presence and expression of *cfr* in VRE, its precise role in conferring resistance to VRE or enterococci isolates remains unclear (Liu et al., 2014; Deshpande et al., 2015). Further study is necessary to better understand the level of resistance conferred by *cfr*(B) in Enterococci.

Tigecycline; a new generation tetracycline offers resistance to the tetracycline class, as a potential option for the treatment of intra-abdominal infections or as a part of combination therapy in bacteremia and infective endocarditis by VRE. Resistance to the tetracycline class is common in Enterococci and VRE, mediated through drug efflux via efflux pumps typically carried on plasmids [*tet*(K), *tet*(L)] or through target protection at the ribosome mediated by *tet*(M), *tet*(O), and *tet*(S) (Miller et al., 2020). We detected *tet*(L) in 81.25% (13/16) and *tet*(M) in 75% (12/16) in the current study. The presence of both the *tet*(L) efflux pump and the *tet*(M) protection factor was associated with resistance in clinical isolates of *E. faecium* (Miller et al., 2020).

The VREfm isolates in the current study also carried a gene for resistance to aminoglycoside, specifically *aac(6′)-li* (100%, 16/16), *ant(6)-Ia* (93.75%, 15/16)*, aph(3′)*-III (100%, 15/16), *aac(6′)-aph(2″)* (*81.25% 13/16, aph(2″)-la* and *ant(9)-la* (6.25%, 1/16)), the macrolide resistance gene, specifically *erm(B)* and *msr(C)* (100%, 16/16)*, erm(T)* (62.5%, 10/16), and *erm(A)* (6.25%, 1/16), the fosfomycin resistance gene, specifically (*fosB,* 6.25%, 1/16), the trimethoprim resistance gene, specifically *dfrG* (18.75%, 3/16), and the clindamycin resistance gene, specifically *lnu*(B) (12.5%, 2/16). Genes involved in vancomycin resistance, namely the *vanA* gene cluster (*van*R, *van*S, *van*H, *van*X, *van*Y, and *van*Z) were present in all VREfms.

**Figure 1:**
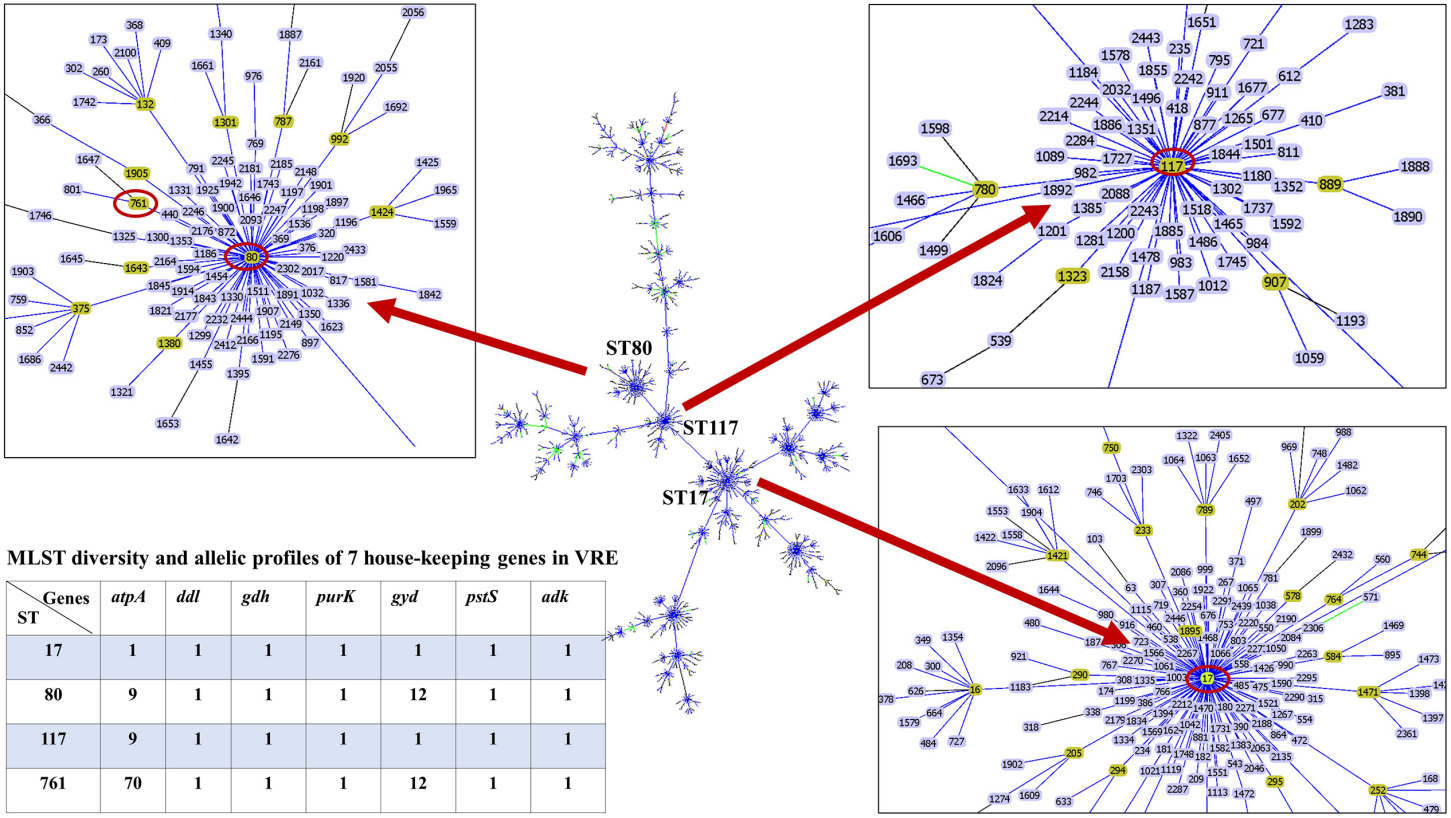
Minimum spanning tree of sequence types (ST) of *E. faecium*, constructed with goeBURST. The 16 VREfm strains belonging to four STs (ST17, ST80, ST117, and ST761) are denoted as red circles.

**Figure 2:**
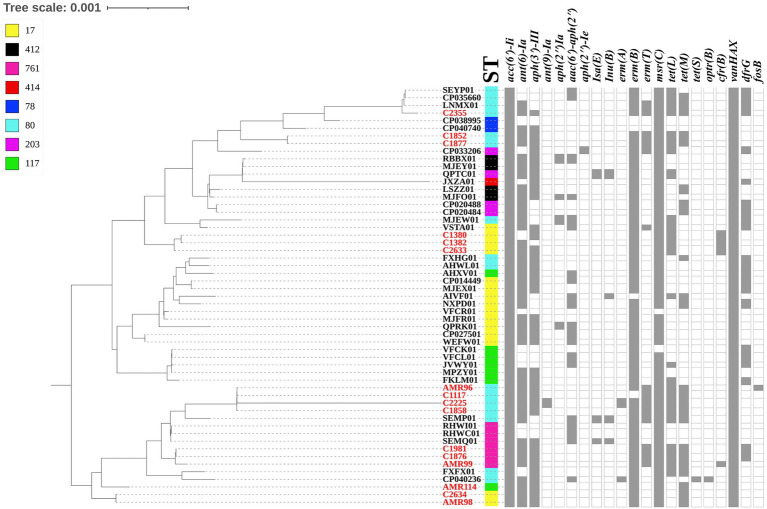
Dendrogram representing the phylogenetic analysis of the VREfm strains generate by Realphy and visualized with interactive tree of life tool. The whole genome sequence of VREfm in our studies is shown in red color. Sequence type (STs) and antibiotic-resistant genes are shown in each isolate. The filled symbols (gray box) reveal the presentation of the genes, whereas unfilled symbols reveal their absence.

The whole genome of the 16 VREfm stains varied from 2.88 to 3.5 Mb. In total, 4 different MLSTs were identified in these 16 VREfm including 7 isolates belonging to ST80, five isolates belonging to ST17, three isolates belonging to ST761, and one isolate belonging to ST117. The goeBURST analysis displayed a clonal complex of VREfm, as shown in Figure 1. ST17 was related to ST117, whereas ST80 was closely related to ST761. A phylogenetic tree was constructed using four STs, as shown in Figure 2. All VREfm ST isolates in this study belonged to CC17, which is a major group of genetic lineages of *E. faecium* that are distributed worldwide and associated with hospital outbreaks (Akpaka et al., 2017). CC17 was divided into clade A, associated with hospital-associated HA *E. faecium*, while clade B was community associated (Gorrie et al., 2019). Clade A has been divided into clade A1 (human clinical strains) and clade A2 (animal derived strains), with the *E. faecium* strains belonging to clade A1 lineage being characterized as resistant to ampicillin and quinolone (Zhou et al., 2020). Similarly, the VREfm isolates in the current study were resistant to ampicillin and quinolone, indicating a human-origin lineage. VREfm belonging to ST17 and ST80 have been reported worldwide, including in Germany (Neumann et al., 2020), France (Sassi et al., 2019), Australia (Lee et al., 2020), Libya (Ahmed et al., 2020), and Thailand, having been isolated from placental tissue, urine, blood, and rectal swabs (Wongnak et al., 2021; Pongchaikul et al., 2023). In Asia, ST17 and ST80 have been reported in China (Sun et al., 2019), India (Bakthavatchalam et al., 2022), and Taiwan (Kuo et al., 2018), whereas an outbreak of VREfm ST761 has been reported in France (Kamus et al., 2022), while ST117 has been identified in Denmark (Pinholt et al., 2019), Greece (Papagiannitsis et al., 2017), and Germany (Xanthopoulou et al., 2020). However, the current study is the first to report VREfm ST761 and ST117 in Thailand.

Two virulence factors were found in all VREfm samples, namely *acm* (collagen adhesin) that contributes to biofilm formation (Gao et al., 2018) and *efaAfm* (cell wall adhesin) (Eaton and Gasson, 2001), while *hylEfm* (glycosyl hydrolase), which affects intestinal colonization and invasive diseases (Panesso et al., 2011), was present in two VREfm isolates of ST80, Additionally, 11 types of plasmid replicons were identified in the analyzed genomes, with rep17 and repUS15 in all VREfm isolates, followed by rep2 (93.75%, 15/16), rep11a (87.5%, 14/16), repUS43 (81.25%, 13/16), repUS12 (62.5%, 10/16), rep18b (43.75%,7/16), repUS7 (31.25%, 5/16), rep14a (25% 4/16), rep1 (12.5%, 2/16), and rep18a (6.25%, 1/16). A limitation of short read sequencing is that it cannot be used to reconstruct individual plasmids, while they often contain repetitive elements, such as IS, which cannot be over spanned by short reads.

**Figure 3:**
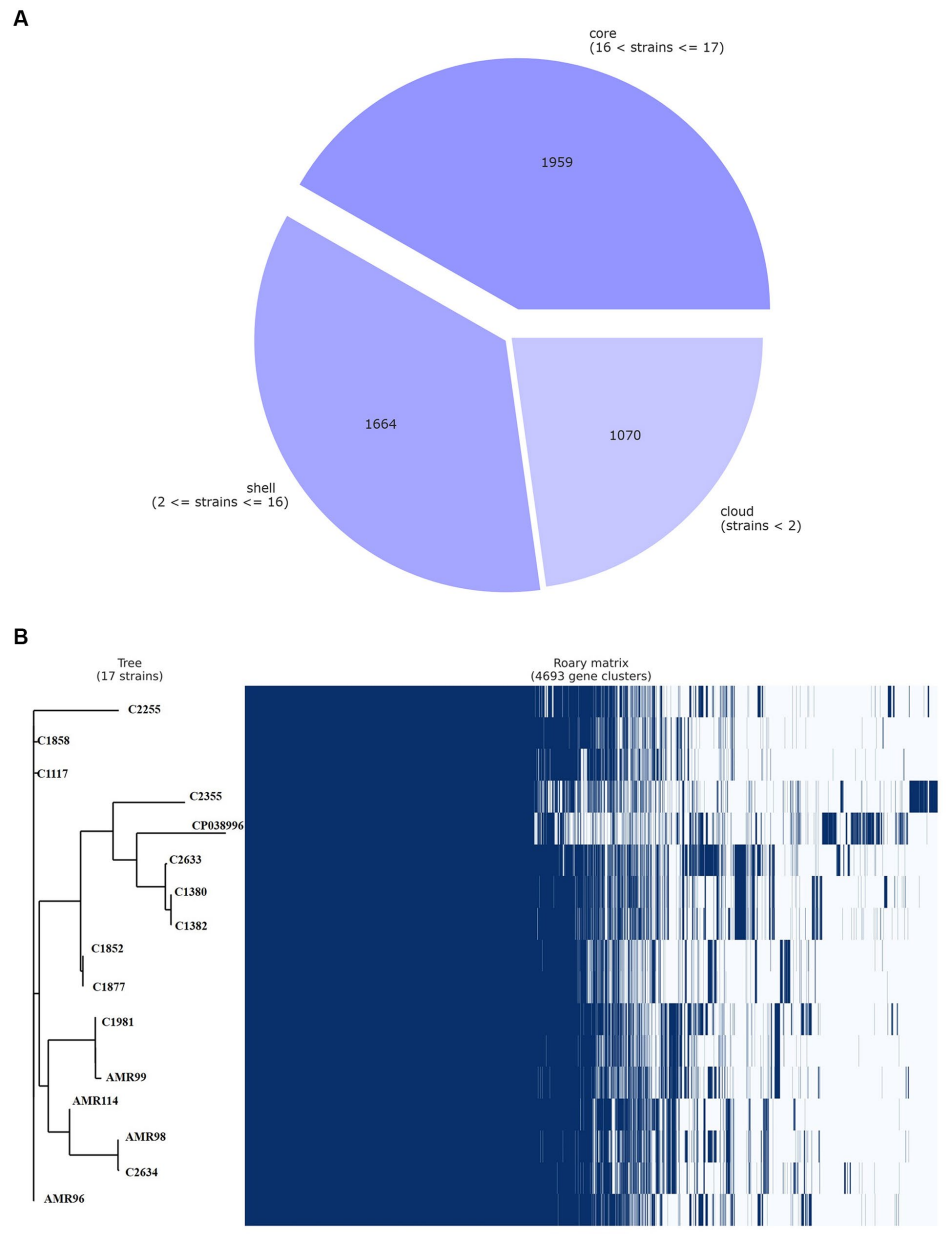
Genetic relatedness analysis of VREfm strains. Visualization of pangenome analysis by Roary software of 17 VREfm strains. **(A)** A pie chart showing the proportion of protein-coding genes in the core, soft-core, shell, and cloud of the pangenome. **(B)** Gene presence/absence matrix showing the distribution of genes in each genome. The phylogenetic tree is based on all of the core gene sequences of the 17 VREfm genomes. Each row corresponds to a branch of the tree. Each column represents an orthologous gene family. Dark blue bar indicates the presence of a gene, and the white bar indicate the absence of a gene.

The pan-genome of VREfm were inferred with Roary, which produced a total of 4,693 genes sequence clusters. The “core genome,” consisting of genes present in all strains was represented by 1,959 genes, accounting for 41.74% of all genes. The remaining 2,734, non-core gens were divided into 1,664 (35.46%) “shell genes” and 1,070 (22.80%) “cloud genes” (see Figure 3A). A heatmap was drawn to visualize the presence or absence of all 4,693 genes. Figure 3B shows a comparison of the phylogenetic tree and a matrix generated both with and without the core and accessory genes of all VREfm.

## 4 Conclusion

This study revealed that all urinary VREfm isolates belonged to CC17. They showed resistance to many antibiotics that harbored numerous antimicrobial-resistant genes, including *van*A, *tet(L)*, *tet(M)*, *aac(*6′*)-li*, *ant(*6*)-Ia*, *aph(*3′*)*-III, *aac(*6′*)-aph(*2″*), aph(*2″*)-la*, *ant(*9*)-la*, *erm(B)*, *msr(C), erm(T)*, *erm(A), fosB, dfrG*, and *cfr(B)*. However, all isolates remained susceptible to chloramphenicol, daptomycin, and linezolid. This highlights the urgent need of rigorous enforcement of infection control measures, in-depth epidemiological analysis by molecular tools for monitoring this evolving threat.

## Data availability statement

The original contributions presented in the study are included in the article, further inquiries can be directed to the correspondingauthor.

## Author contributions

PC: Conceptualization, Writing – original draft. PB: Formal analysis, Writing – review & editing. PJ: Investigation, Methodology, Software, Writing – review & editing. TW: Investigation, Methodology, Software, Writing – review & editing. RH: Formal analysis, Writing – review & editing. AK: Conceptualization, Resources, Supervision, Writing – review & editing. NS: Conceptualization, Funding acquisition, Writing – original draft, Writing – review & editing.

## Footnotes

^1^ https://www.bioinformatics.babraham.ac.uk/projects/fastqc/

^2^ http://bacdb.cn/BacWGSTdb/

^3^ http://realphy.unibas.ch

^4^ http://itol.embl.de

## References

[ref1] Ahmed M. O. Baptiste K. E. (2018). Vancomycin-resistant enterococci: a review of antimicrobial resistance mechanisms and perspectives of human and animal health. Microb. Drug Resist. 24, 590–606. doi: 10.1089/MDR.2017.0147, PMID: 29058560

[ref2] Ahmed M. O. Elramalli A. K. Baptiste K. E. Daw M. A. Zorgani A. Brouwer E.. (2020). Whole genome sequence analysis of the first vancomycin-resistant *Enterococcus faecium* isolates from a Libyan hospital in Tripoli. Microb. Drug Resist. 26, 1390–1398. doi: 10.1089/MDR.2019.0095, PMID: 32181678

[ref3] Akpaka P. E. Kissoon S. Wilson C. Jayaratne P. Smith A. Golding G. R. (2017). Molecular characterization of vancomycin-resistant *Enterococcus faecium* isolates from Bermuda. PLoS One 12:e0171317. doi: 10.1371/JOURNAL.PONE.0171317, PMID: 28267763 PMC5340350

[ref4] Alcock B. P. Huynh W. Chalil R. Smith K. W. Raphenya A. R. Wlodarski M. A.. (2023). CARD 2023: expanded curation, support for machine learning, and resistome prediction at the comprehensive antibiotic resistance database. Nucleic Acids Res. 51, D690–D699. doi: 10.1093/NAR/GKAC920, PMID: 36263822 PMC9825576

[ref5] Bakthavatchalam Y. D. Puraswani M. Livingston A. Priya M. Venkatesan D. Sharma D.. (2022). Novel linear plasmids carrying vanA cluster drives the spread of vancomycin resistance in *Enterococcus faecium* in India. J. Glob. Antimicrob. Resist. 29, 168–172. doi: 10.1016/J.JGAR.2022.03.013, PMID: 35339734

[ref6] Benamrouche N. Guettou B. Henniche F. Z. Assaous F. Laouar H. Ziane H.. (2021). Vancomycin-resistant *Enterococcus faecium* in Algeria: phenotypic and genotypic characterization of clinical isolates. J. Infect. Dev. Ctries. 15, 95–101. doi: 10.3855/JIDC.12482, PMID: 33571151

[ref7] Bertels F. Silander O. K. Pachkov M. Rainey P. B. Van Nimwegen E. (2014). Automated reconstruction of whole-genome phylogenies from short-sequence reads. Mol. Biol. Evol. 31, 1077–1088. doi: 10.1093/MOLBEV/MSU088, PMID: 24600054 PMC3995342

[ref8] Bortolaia V. Kaas R. S. Ruppe E. Roberts M. C. Schwarz S. Cattoir V.. (2020). ResFinder 4.0 for predictions of phenotypes from genotypes. J. Antimicrob. Chemother. 75, 3491–3500. doi: 10.1093/JAC/DKAA345, PMID: 32780112 PMC7662176

[ref9] Carattoli A. Hasman H. (2020). PlasmidFinder and *in silico* pMLST: identification and typing of plasmid replicons in whole-genome sequencing (WGS). Methods Mol. Biol. 2075, 285–294. doi: 10.1007/978-1-4939-9877-7_20, PMID: 31584170

[ref10] CLSI (2022). Performance standards for antimicrobial susceptibility testing. CLSI Document M100–S32, 32th Edn. Wayne, PA: Clinical and Laboratory Standards Institute, USA.

[ref12] Deshpande L. M. Ashcraft D. S. Kahn H. P. Pankey G. Jones R. N. Farrell D. J.. (2015). Detection of a New cfr-Like Gene, cfr(B), in *Enterococcus faecium* isolates recovered from human specimens in the united states as part of the SENTRY antimicrobial surveillance program. Antimicrob. Agents Chemother. 59, 6256–6261. doi: 10.1128/AAC.01473-15, PMID: 26248384 PMC4576063

[ref13] Eaton T. J. Gasson M. J. (2001). Molecular screening of *Enterococcus* virulence determinants and potential for genetic exchange between food and medical isolates. Appl. Environ. Microbiol. 67, 1628–1635. doi: 10.1128/AEM.67.4.1628-1635.2001, PMID: 11282615 PMC92779

[ref15] EUCAST (2021). The European Committee on Antimicrobial Susceptibility Testing. Breakpoint tables for interpretation of MICs and zone diameters. Version 11.0, 2021. Available at: http://www.eucast.org.

[ref16] Fallah F. Yousefi M. Pourmand M. R. Hashemi A. Nazari Alam A. Afshar D. (2017). Phenotypic and genotypic study of biofilm formation in Enterococci isolated from urinary tract infections. Microb. Pathog. 108, 85–90. doi: 10.1016/J.MICPATH.2017.05.014, PMID: 28483600

[ref17] Feng Y. Zou S. Chen H. Yu Y. Ruan Z. (2021). BacWGSTdb 2.0: a one-stop repository for bacterial whole-genome sequence typing and source tracking. Nucleic Acids Res. 49, D644–D650. doi: 10.1093/NAR/GKAA821, PMID: 33010178 PMC7778894

[ref18] Francisco A. P. Vaz C. Monteiro P. T. Melo-Cristino J. Ramirez M. Carriço J. A. (2012). PHYLOViZ: Phylogenetic inference and data visualization for sequence based typing methods. BMC Bioinformatics 13, 1–10. doi: 10.1186/1471-2105-13-87/FIGURES/5 22568821 PMC3403920

[ref19] Gao W. Howden B. P. Stinear T. P. (2018). Evolution of virulence in *Enterococcus faecium*, a hospital-adapted opportunistic pathogen. Curr. Opin. Microbiol. 41, 76–82. doi: 10.1016/J.MIB.2017.11.030, PMID: 29227922

[ref20] Gorrie C. Higgs C. Carter G. Stinear T. P. Howden B. (2019). Genomics of vancomycin-resistant *Enterococcus faecium*. MGen 5:e000283. doi: 10.1099/MGEN.0.000283, PMID: 31329096 PMC6700659

[ref21] Gozalan A. Coskun-Ari F. F. Ozdem B. Unaldi O. Celikbilek N. Kirca F.. (2015). Molecular characterization of vancomycin-resistant *Enterococcus faecium* strains isolated from carriage and clinical samples in a tertiary hospital, Turkey. J. Pathol. Bacteriol. 64, 759–766. doi: 10.1099/JMM.0.000088, PMID: 25976005

[ref22] Jackson C. R. Fedorka-Cray P. J. Barrett J. B. (2004). Use of a genus- and species-specific multiplex pcr for identification of enterococci. J. Clin. Microbiol. 42, 3558–3565. doi: 10.1128/JCM.42.8.3558-3565.2004, PMID: 15297497 PMC497640

[ref23] Kamus L. Auger G. Gambarotto K. Houivet J. Ramiandrisoa M. Picot S.. (2022). Investigation of a vanA linezolid-and vancomycin-resistant *Enterococcus faecium* outbreak in the Southwest Indian Ocean (Reunion Island). Int. J. Antimicrob. Agents 60:106686. doi: 10.1016/J.IJANTIMICAG.2022.106686, PMID: 36503708

[ref24] Kuo A. J. Shu J. C. Liu T. P. Lu J. J. Lee M. H. Wu T. S.. (2018). Vancomycin-resistant *Enterococcus faecium* at a university hospital in Taiwan, 2002-2015: Fluctuation of genetic populations and emergence of a new structure type of the Tn1546-like element. J. Microbiol. Immunol. Infect. 51, 821–828. doi: 10.1016/J.JMII.2018.08.008 30201132

[ref25] Lang J. Zhu R. Sun X. Zhu S. Li T. Shi X.. (2021). Evaluation of the MGISEQ-2000 sequencing platform for illumina target capture sequencing libraries. Front. Genet. 12:730519. doi: 10.3389/FGENE.2021.730519, PMID: 34777467 PMC8578046

[ref26] Larsen M. V. Cosentino S. Rasmussen S. Friis C. Hasman H. Marvig R. L.. (2012). Multilocus sequence typing of total-genome-sequenced bacteria. J. Clin. Microbiol. 50, 1355–1361. doi: 10.1128/JCM.06094-11, PMID: 22238442 PMC3318499

[ref27] Lee T. Pang S. Stegger M. Sahibzada S. Abraham S. Daley D.. (2020). A three-year whole genome sequencing perspective of *Enterococcus faecium* sepsis in Australia. PLoS One 15:e0228781. doi: 10.1371/JOURNAL.PONE.0228781, PMID: 32059020 PMC7021281

[ref28] Letunic I. Bork P. (2021). Interactive Tree Of Life (iTOL) v5: an online tool for phylogenetic tree display and annotation. Nucleic Acids Res. 49, W293–W296. doi: 10.1093/NAR/GKAB301, PMID: 33885785 PMC8265157

[ref29] Li W. Hu J. Li L. Zhang M. Cui Q. Ma Y.. (2022). New mutations in cls lead to daptomycin resistance in a clinical vancomycin-and daptomycin-resistant *Enterococcus faecium* strain. Front. Microbiol. 13:896916. doi: 10.3389/FMICB.2022.896916 35801099 PMC9253605

[ref30] Linden P. K. (2002). Treatment options for vancomycin-resistant enterococcal infections. Drugs 62, 425–441. doi: 10.2165/00003495-200262030-00002/METRICS 11827558

[ref31] Liu L. Coenye T. Burns J. L. Whitby P. W. Stull T. L. LiPuma J. J. (2002). Ribosomal DNA-directed PCR for identification of Achromobacter (Alcaligenes) xylosoxidans recovered from sputum samples from cystic fibrosis patients. J. Clin. Microbiol. 40, 1210–1213. doi: 10.1128/JCM.40.4.1210-1213.2002, PMID: 11923333 PMC140369

[ref32] Liu Y. Wang Y. Schwarz S. Wang S. Chen L. Wu C.. (2014). Investigation of a multiresistance gene cfr that fails to mediate resistance to phenicols and oxazolidinones in *Enterococcus faecalis*. J. Antimicrob. Chemother. 69, 892–898. doi: 10.1093/JAC/DKT459, PMID: 24272266

[ref33] Malberg Tetzschner A. M. Johnson J. R. Johnston B. D. Lund O. Scheutz F. (2020). *In silico* genotyping of *Escherichia coli* isolates for extraintestinal virulence genes by use of whole-genome sequencing data. J. Clin. Microbiol. 58:e01269-20. doi: 10.1128/JCM.01269-20, PMID: 32669379 PMC7512150

[ref34] Miller W. R. Murray B. E. Rice L. B. Arias C. A. (2020). Resistance in vancomycin-resistant enterococci. Infect. Dis. Clin. N. Am. 34, 751–771. doi: 10.1016/J.IDC.2020.08.004, PMID: 33131572 PMC7640809

[ref35] National Antimicrobial Resistance Surveillance Thailand (2022). NARST. Available at: http://narst.dmsc.moph.go.th/ (Accessed August 1, 2023).

[ref36] Neumann B. Bender J. K. Maier B. F. Wittig A. Fuchs S. Brockmann D.. (2020). Comprehensive integrated NGS-based surveillance and contact-network modeling unravels transmission dynamics of vancomycin-resistant enterococci in a high-risk population within a tertiary care hospital. PLoS One 15:e0235160. doi: 10.1371/JOURNAL.PONE.0235160, PMID: 32579600 PMC7314025

[ref001] Page A. J. Cummins C. A. Hunt M. Wong V. K. Reuter S. Holden M. T. G.. (2015). Roary: rapid large-scale prokaryote pan genome analysis. Bioinformatics. 31:3691–3693. doi: 10.1093/bioinformatics/btv421, PMID: 26198102 PMC4817141

[ref37] Panesso D. Montealegre M. C. Rincón S. Mojica M. F. Rice L. B. Singh K. V.. (2011). The hylEfm gene in pHylEfm of *Enterococcus faecium* is not required in pathogenesis of murine peritonitis. BMC Microbiol. 11:20. doi: 10.1186/1471-2180-11-20, PMID: 21266081 PMC3039558

[ref38] Papagiannitsis C. C. Malli E. Florou Z. Medvecky M. Sarrou S. Hrabak J.. (2017). First description in Europe of the emergence of *Enterococcus faecium* ST117 carrying both vanA and vanB genes, isolated in Greece. J. Glob. Antimicrob. Resist. 11, 68–70. doi: 10.1016/J.JGAR.2017.07.010, PMID: 28754459

[ref39] Pérez-Hernández X. Méndez-Álvarez S. Claverie-Martín F. (2002). A PCR assay for rapid detection of vancomycin-resistant enterococci. Diagn. Microbiol. Infect. Dis. 42, 273–277. doi: 10.1016/S0732-8893(01)00360-1 12007446

[ref40] Pinholt M. Bayliss S. C. Gumpert H. Worning P. Jensen V. V. S. Pedersen M.. (2019). WGS of 1058 *Enterococcus faecium* from Copenhagen, Denmark, reveals rapid clonal expansion of vancomycin-resistant clone ST80 combined with widespread dissemination of a vanA-containing plasmid and acquisition of a heterogeneous accessory genome. J. Antimicrob. Chemother. 74, 1776–1785. doi: 10.1093/JAC/DKZ118, PMID: 30929020

[ref41] Pongchaikul P. Romero R. Mongkolsuk P. Vivithanaporn P. Wongsurawat T. Jenjaroenpun P.. (2023). Genomic analysis of *Enterococcus faecium* strain RAOG174 associated with acute chorioamnionitis carried antibiotic resistance gene: is it time for precise microbiological identification for appropriate antibiotic use? BMC Genomics 24:405. doi: 10.1186/S12864-023-09511-1, PMID: 37468842 PMC10354890

[ref42] Rao C. Dhawan B. Vishnubhatla S. Kapil A. Das B. Sood S. (2021). Clinical and molecular epidemiology of vancomycin-resistant *Enterococcus faecium* bacteremia from an Indian tertiary hospital. Eur. J. Clin. Microbiol. Infect. Dis. 40, 303–314. doi: 10.1007/S10096-020-04030-3, PMID: 32909085

[ref43] Saenhom N. Boueroy P. Chopjitt P. Hatrongjit R. Kerdsin A. (2022). Distinguishing clinical *Enterococcus faecium* strains and resistance to vancomycin using a simple in-house screening test. Antibiotics (Basel, Switzerland) 11:286. doi: 10.3390/ANTIBIOTICS11030286 35326750 PMC8944677

[ref44] Sassi M. Guérin F. Zouari A. Beyrouthy R. Auzou M. Fines-Guyon M.. (2019). Emergence of optrA-mediated linezolid resistance in enterococci from France, 2006-16. J. Antimicrob. Chemother. 74, 1469–1472. doi: 10.1093/JAC/DKZ097, PMID: 30897199

[ref45] Shrestha S. Kharel S. Homagain S. Aryal R. Mishra S. K. (2021). Prevalence of vancomycin-resistant enterococci in Asia-A systematic review and meta-analysis. J. Clin. Pharm. Ther. 46, 1226–1237. doi: 10.1111/JCPT.13383, PMID: 33630382

[ref46] Sun H. L. Liu C. Zhang J. J. Zhou Y. M. Xu Y. C. (2019). Molecular characterization of vancomycin-resistant enterococci isolated from a hospital in Beijing, China. J. Microbiol. Immunol. Infect. 52, 433–442. doi: 10.1016/J.JMII.2018.12.008, PMID: 30827858

[ref47] Tacconelli E. Carrara E. Savoldi A. Harbarth S. Mendelson M. Monnet D. L.. (2018). Discovery, research, and development of new antibiotics: the WHO priority list of antibiotic-resistant bacteria and tuberculosis. Lancet Infect. Dis. 18, 318–327. doi: 10.1016/S1473-3099(17)30753-3, PMID: 29276051

[ref48] Wongnak K. Pattanachaiwit S. Rattanasirirat W. Limsrivanichakorn S. Kiratisin P. Assanasen S.. (2021). First characterization of Tn1546-like structures of vancomycin-resistant *Enterococcus faecium* Thai isolates. J. Infect. Chemother. 27, 991–998. doi: 10.1016/J.JIAC.2021.02.013, PMID: 33663929

[ref49] Xanthopoulou K. Peter S. Tobys D. Behnke M. Dinkelacker A. G. Eisenbeis S.. (2020). Vancomycin-resistant *Enterococcus faecium* colonizing patients on hospital admission in Germany: prevalence and molecular epidemiology. J. Antimicrob. Chemother. 75, 2743–2751. doi: 10.1093/JAC/DKAA271, PMID: 32699884

[ref50] Yang J. X. Li T. Ning Y. Z. Shao D. H. Liu J. Wang S. Q.. (2015). Molecular characterization of resistance, virulence and clonality in vancomycin-resistant *Enterococcus faecium* and *Enterococcus faecalis*: A hospital-based study in Beijing, China. Infect. Genet. Evol. 33, 253–260. doi: 10.1016/J.MEEGID.2015.05.012, PMID: 25976380

[ref51] Zhou X. Willems R. J. L. Friedrich A. W. Rossen J. W. A. Bathoorn E. (2020). *Enterococcus faecium*: from microbiological insights to practical recommendations for infection control and diagnostics. Antimicrob. Resist. Infect. Control 9:130. doi: 10.1186/S13756-020-00770-1, PMID: 32778149 PMC7418317

